# Damage-free light-induced assembly of intestinal bacteria with a bubble-mimetic substrate

**DOI:** 10.1038/s42003-021-01807-w

**Published:** 2021-03-22

**Authors:** Kota Hayashi, Yasuyuki Yamamoto, Mamoru Tamura, Shiho Tokonami, Takuya Iida

**Affiliations:** 1 Department of Physical Science, Graduate School of Science, Osaka Prefecture University, Osaka, Japan; 2 Research Institute for Light-induced Acceleration System (RILACS), Osaka Prefecture University, Osaka, Japan; 3 Department of Applied Chemistry, Graduate School of Engineering, Osaka Prefecture University, Osaka, Japan

**Keywords:** Assay systems, Symbiosis

## Abstract

Rapid evaluation of functions in densely assembled bacteria is a crucial issue in the efficient study of symbiotic mechanisms. If the interaction between many living microbes can be controlled and accelerated via remote assembly, a cultivation process requiring a few days can be ommitted, thus leading to a reduction in the time needed to analyze the bacterial functions. Here, we show the rapid, damage-free, and extremely dense light-induced assembly of microbes over a submillimeter area with the “bubble-mimetic substrate (BMS)”. In particular, we successfully assembled 10^4^–10^5^ cells of lactic acid bacteria (*Lactobacillus casei*), achieving a survival rate higher than 95% within a few minutes without cultivation process. This type of light-induced assembly on substrates like BMS, with the maintenance of the inherent functions of various biological samples, can pave the way for the development of innovative methods for rapid and highly efficient analysis of functions in a variety of microbes.

## Abstract (web summary)

Hayashi et al. propose a method to realise rapid, damage-free, remote light-induced assembly of microbes over a submillimeter area with the “bubble-mimetic substrate”. They assemble 10^4^–10^5^ cells of lactic acid bacteria with a survival rate higher than 95% around a laser spot. This technique is promising in developing methods for rapid and high-efficiency functional analysis of microbes.

## Introduction

Bacterial symbiotic mechanism is a crucial issue for human health and environmental problems; for instance, quorum sensing^1–7^ and probiotics of microbes^8–12^ have attracted significant attention. Quorum sensing is known as a mechanism arising from the interaction between bacteria, where bacteria sense the cell population density via the concentration of signaling molecules and determine genetic expression. For example, such a quorum sensing is considered to be the cause of squid luminescence arising from luminescent bacteria^3^, and the cause of infection arising from opportunistic bacterium (*Pseudomonas aeruginosa* etc.)^4,5^. The opportunistic bacterium does not express pathogenicity at low concentration, but does when the immune system of the host is weakened or its concentration increases significantly to emit the pathogen. There are on-going studies on inhibiting quorum sensing in relation to drug development to restrain pathogenicity^6,7^. Especially, the relationship between intestinal flora and human health has been actively studied, and it has been reported that probiotics by lactic acid bacteria such as *Lactobacillus* sp. play an important role in controlling allergic diseases^8–12^. As the concentration of signaling molecules used in quorum sensing is considered to be too low to be detected (of the order of nM) by general analytic methods (e.g., NMR), the use of bacteria as reporter strains that luminesce comprises the main analytical method, wherein signaling molecules are added to a sampling liquid containing bacteria and cultivated over a 1-day period and quorum sensing is investigated visually after such a procedure^13^.

If live bacteria can be assembled rapidly and densely, the interaction between many microbes, including the process of quorum sensing, can be controlled and accelerated, thus leading to the reduction in the time of analyzing the bacterial functions omitting a cultivation process requiring a few days. For example, the use of an optical tweezer is known as a method for trapping and gathering cells and microbes using electromagnetic and non-thermal light-induced forces^14^. This method is effective for precisely manipulating spatial position and motions of a few biological samples, and previously, optical trapping of a few cells using a metallic nanoantenna^15^, optical manipulation of organelles and molecules in a cell^16^, and trapping of a few molecules in a nanogap^17^ using optical tweezers have been demonstrated.

If the condensation of many bacteria or biological nanomaterials could be remotely controlled rapidly at a large scale, bacterial interaction can be investigated effectively. For example, photothermal effect can be used as a tool for generating macroscopic flow to transport small objects under appropriate liquid phase control. However, in some studies, photothermal effect induced by laser light irradiation on metallic nanostructures was used to destroy biological cells^18–23^. On the other hand, paying attention to the collective phenomenon of localized surface plasmons, the principles of light-induced acceleration of the biochemical reactions of biological nanomaterials (e.g., DNA and proteins) were realized based on the synergetic effect of light-induced force and light-induced convection due to photothermal effect^24–27^. In previous studies, “photothermal assembly” based on convection due to photothermal effect and the formation of a submillimeter bubble under local high-temperature conditions were used for large-scale production of organic molecular poly-crystals^28^, electrical detection of bio-nanomaterials^29^, and rapid trapping of microbes at a high density^30,31^. As fundamental studies, micron-sized structures have been formed by assembling nanoparticles using a laser-induced microbubble^32,33^, and the convection mechanism around the photothermal microbubble has been investigated in detail^34,35^. Further, a phenomenon called superheating was used for bubble generation by heating a liquid to more than 100 °C^36^. It is suspected that this high temperature may be an obstacle to the evaluation of biological functions, such as the interaction of microbes, because the samples can be damaged by heat when the biological samples are used as dispersoids. To overcome this obstacle, several strategies have been suggested to develop microstructured substrates by arranging periodic microstructures and random nanostructures on the substrate surface by self-organization to effectively generate light-induced force and light-induced convection^37–39^, and bacteria can be assembled on them with high survival rates due to the honeycomb-shaped microstructures on the substrate surface^40^. In this context, we focused on a ring-shaped stagnant area formed between the bubble and the substrate as a trapping site for avoiding the photothermal effect on biological dispersoids.

Here, we present a substrate to effectively generate light-induced convection without thermal damage to dispersoids by optical design and spatial configuration of the heat source for achieving photothermal assembly of bacteria. In particular, in order to develop a method for achieving highly dense, large-scale, and damage-free light-induced assembly of dispersoids and to clarify the underlying physicochemical mechanism of the assembly, we attempted to produce a bubble-mimetic substrate consisting of a polystyrene particle (an imitation bubble as large as a submillimeter light-induced bubble) fixed on a glass substrate. Using this substrate, we performed experiments to remotely control the assembly of lactic acid bacteria, *Lactobacillus casei* (*L. casei*), as an example of intestinal bacteria, as biological dispersoids by means of laser irradiation.

## Results

**Fig. 1:**
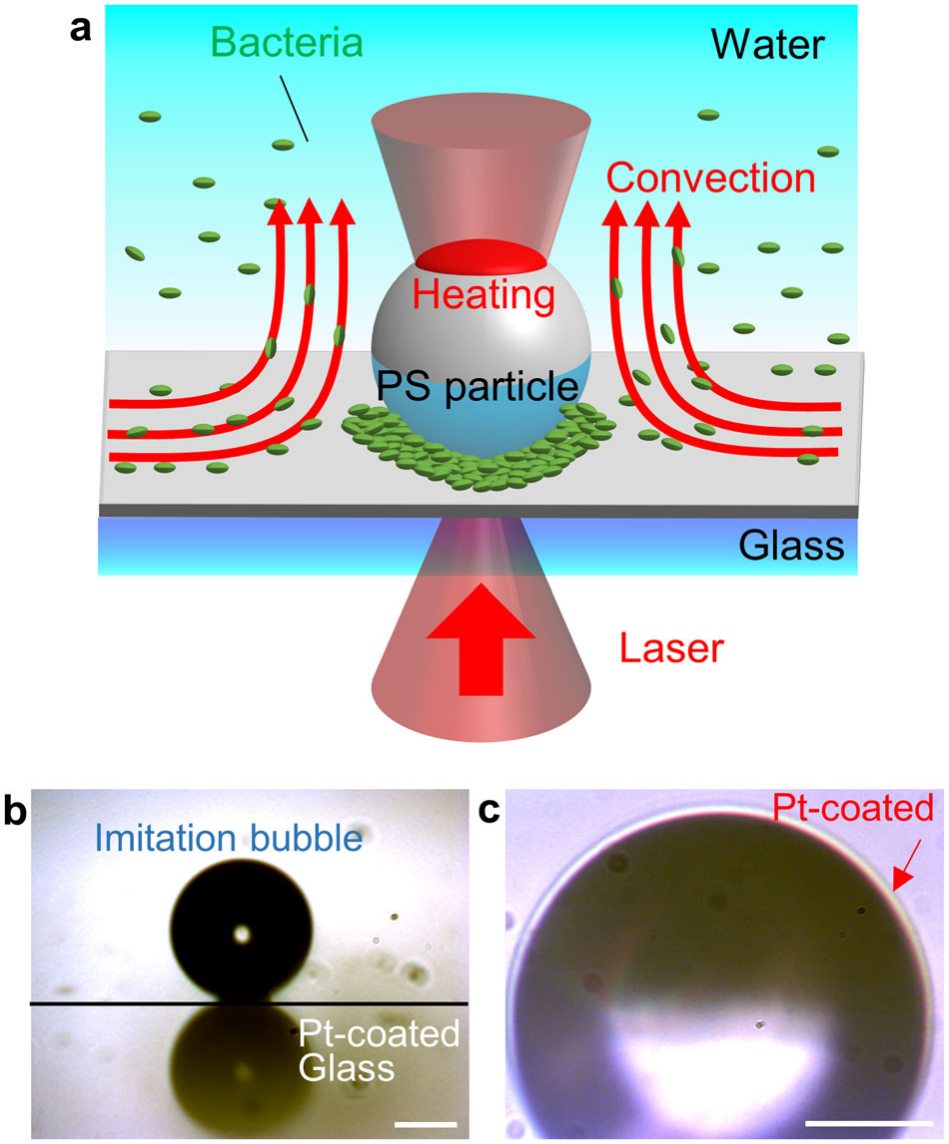
Light-induced assembly of bacteria on a bubble-mimetic substrate (BMS). **a** Schematic illustration of light-induced assembly of bacteria on a BMS. **b** Optical transmission images of the side-view of an imitation bubble. **c** Pt-coated upper region of an imitation bubble absorbed white light from halogen lump through an enlarged transmission image. Scale bar is 45 μm in **b**, and 30 μm in **c**.

We prepared a bubble-mimetic substrate (BMS), consisting of a polystyrene particle as an imitation bubble of 100 µm diameter chemically fixed on a glass substrate, and subsequently coated a platinum thin film (thickness: 10 nm) on the substrate as a heat source of light-induced assembly, as shown in Fig. 1a. Figure 1b, c show the side-view and enlarged transmission images, respectively. We irradiated the platinum thin film on the imitation bubble with a near-infrared continuous wave laser (wavelength: 1064 nm), which was focused on the substrate surface using an objective lens (×40, NA = 0.6) for 300 s. *L. casei* (concentrations: 1.43 × 10^8^, 7.15 × 10^7^, and 1.43 × 10^7^ cells/mL, scanning electron microscopy image is shown in Supplementary Fig. 1) stained with two fluorescent dyes (SYTO9 and propidium iodide (PI)) were used as dispersoids. A non-ionic surfactant (concentration: 9.04 × 10^−5^ M)^31^ was also included in the dispersion liquid. After laser irradiation with the optical system in Supplementary Fig. 2a, fluorescent images were recorded using a mercury lamp to excite the sample.

**Fig. 2:**
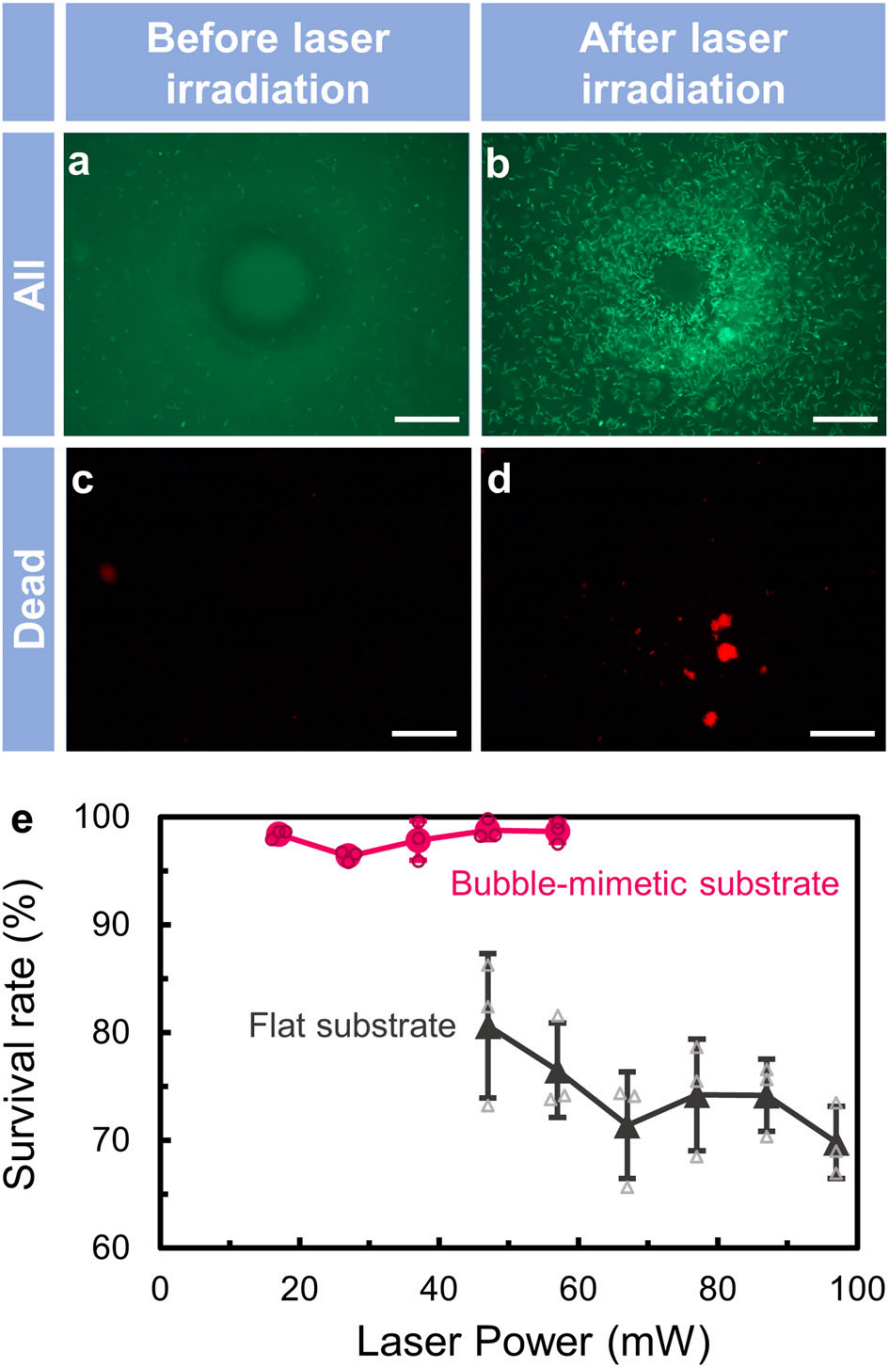
Survival rate of bacteria under laser irradiation. Fluorescent images acquired (**a**, **c**) before and (**b**, **d**) after laser irradiation (*L. casei* concentration: 1.43 × 10^8^ cells/mL). Green fluorescent images (**a**, **b**) show live and dead bacteria while the red fluorescent images (**c**, **d**) show only dead bacteria in the medium; each scale bar is 50 µm. **e** The relationship between the laser power and survival rate on a bubble-mimetic substrate and a flat substrate (bacterial concentration on flat substrate: 2.38 × 10^8^ cells/mL, bacterial concentration on bubble-mimetic substrate: 1.43 × 10^8^ cells/mL) (*n* = 3 independent experiments). The error bars represent the standard deviation. Individual values are shown as small plots.

Figure 2 shows the fluorescent images acquired before and after laser irradiation at 27 mW laser power for the light-induced assembly of *L. casei* (concentration: 1.43 × 10^8^ cells/mL) on BMS. Figure 2a, b show live and dead bacteria, and Fig. 2c, d show dead bacteria. Particularly, Fig. 2b reveals that the bacteria were assembled under laser irradiation. As demonstrated in Supplementary Movie 1, *L. casei* were transported toward the imitation bubble by convection generated just after laser irradiation at 27 mW power, where they assembled around the imitation bubble at a large scale. The convection stopped when the laser irradiation was stopped. The same phenomenon was observed with other dispersoids such as *Staphylococcus aureus* (*S. aureus*) and polystyrene particles (see Supplementary Figs. 4 and 5).

The number of assembled bacteria (*N*_AB_) and the survival rate of bacteria can be estimated from the fluorescent images in Fig. 2 (see Methods in detail of estimation). In the case of assembly on BMS, *N*_AB_ was found to be higher than 35,000 cells. As shown in Fig. 2c, d, dead bacteria were found after laser irradiation. However, a high bacterial survival rate of 97% was achieved. Furthermore, we also investigated the relationship between the bacterial survival rate and laser power (see Fig. 2e). The fluorescent images obtained at different laser powers are shown in Supplementary Fig. 3. The survival rate at 1.43 × 10^8^ cells/mL was higher than 95% at all the laser powers on the BMS (see Supplementary Fig. 6 for data obtained at other bacterial concentrations). To compare the survival rate with that obtained under general photothermal assembly using a real bubble, we also estimated the survival rate after laser irradiation at powers ranging from 47 to 97 mW for 300 s on a flat substrate coated with a platinum thin film (thickness: 10 nm). In this case, a laser power of 47 mW is the threshold power required to stably generate a real bubble on the flat substrate, and the concentration of *L. casei* was 2.38 × 10^8^ cells/mL, where the optical transmission and fluorescent images are shown in Supplementary Fig. 7. On the flat substrate, the maximum bacterial survival rate with laser irradiation at 47 mW laser power was found to be ~80%, which is 15% lower than the minimum survival rate achieved with the BMS. In addition, the survival rate of bacteria on the flat substrate decreased from 80 to 70% with an increase in laser power. These results reveal the capability of BMS in facilitating the assembly of bacteria without thermal damage. Moreover, in the case of *S*. *aureus* assembled by using BMS with a formed gold thin film, the average *N*_AB_ was ~4700 cells and the average survival rate was 88.3%. This result indicates that BMS can be applied to other biological samples and metallic coating.

**Fig. 3:**
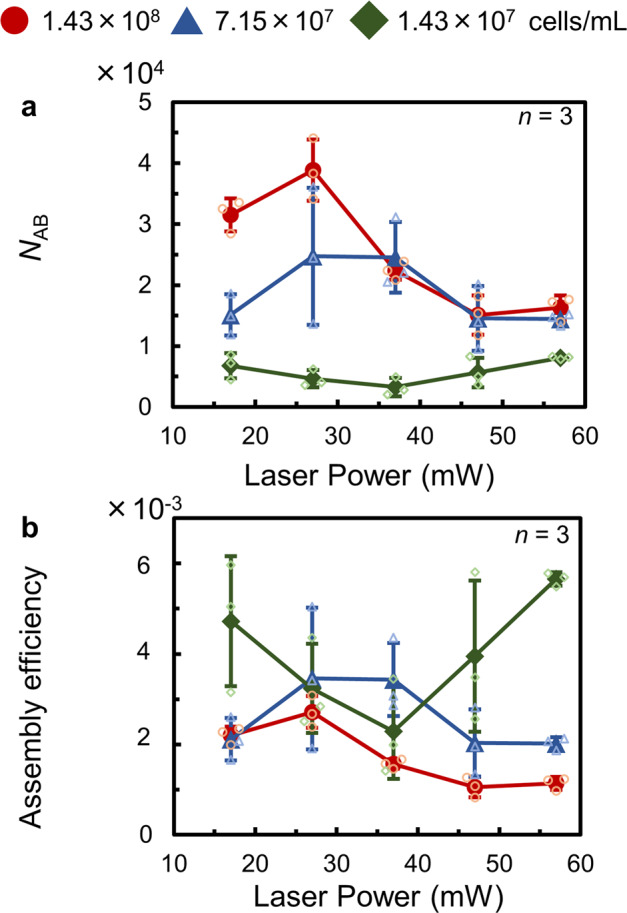
Relationship between the laser power and number of assembled bacteria (*N*_AB_) or assembly efficiency at each bacterial concentration studied. **a** Dependence of *N*_AB_ on laser power (*n* = 3 independent experiments). **b** Dependence of the assembly efficiency of the bacteria on laser power (*n* = 3 independent experiments). The error bars represent the standard deviation. Individual values are shown as small plots.

We further investigated the relationship between *N*_AB_ and laser power at each bacterial concentration (1.43 × 10^8^, 7.15 × 10^7^, and 1.43 × 10^7^ cells/mL). The data for BMS are shown in Fig. 3a and that for the flat substrate is shown in Supplementary Fig. 8. *N*_AB_ increased up to a laser power of 27 mW at 1.43 × 10^8^ and 7.15 × 10^7^ cells/mL. However, *N*_AB_ began to decrease at 37 and 47 mW laser power, respectively, at 1.43 × 10^8^ cells/mL and 7.15 × 10^7^ cells/mL. As higher laser power generates faster convection, we expected the assembly of more dispersoids. However, the results obtained indicate that *N*_AB_ decreases at too high a laser power. This is caused by the shrinkage of the stagnant area owing to the fast convection around the bubble. In addition, *N*_AB_ was almost same at both 1.43 × 10^8^ cells/mL and 7.15 × 10^7^ cells/mL at a laser power of higher than 37 mW, because the stagnant area formed around the imitation bubble is considered to be independent of the concentration of dispersoids. At 1.43 × 10^7^ cells/mL, *N*_AB_ was low even upon increasing the laser power, which is different from the results obtained with 1.43 × 10^8^ cells/mL and 7.15 × 10^7^ cells/mL. This tendency is due to the initial low concentration of the bacteria, owing to which the number of dispersoids around the imitation bubble before laser irradiation was much lower than those at other concentrations. From these results, 27 mW of laser power was determined to be the optimal value for the assembly of *L. casei* within this concentration regime. Figure 3b shows the relationship between the laser power and assembly efficiency, which is *N*_AB_ over the total number of bacteria in the dispersion liquid. The assembly efficiency was found to be inversely proportional to the concentration of bacteria, indicating that the dispersoids can be trapped more efficiently at a low concentration. Thus, bacterial assembly can be achieved even at a low initial concentration of bacteria in the dispersion liquid.

**Fig. 4:**
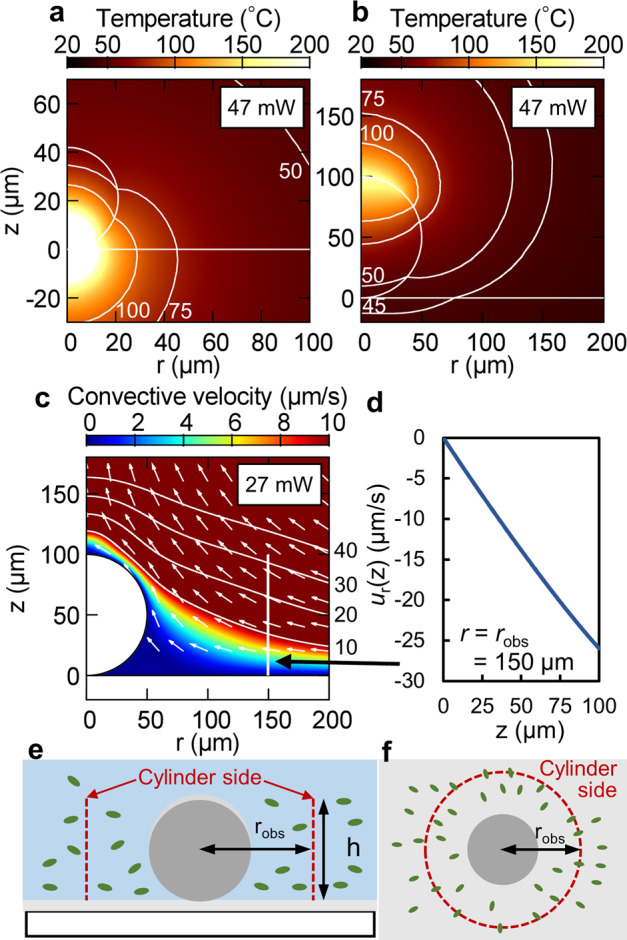
Simulation of light-induced assembly with bubble-mimetic substrate. The distribution of (**a**, **b**) temperature and (**c**) convective velocity. Results of simulation for (**a**) a flat substrate and (**b**, **c**) bubble-mimetic substrate at a laser power of (**a**, **b**) 47 mW and (**c**) 27 mW. The white lines show the contour of the temperature, 45, 50, 75, and 100 °C in **a** and **b**, and velocity, 10, 20, 30, and 40 µm/s in **c**. **d** *r*-component of the convective velocity *u*_r_(z) at the white vertical line in **c**; *r* = *r*_obs_ = 150 µm. **e** Top view and (**f**) side view of the cylindrical model used to estimate the number of transported bacteria.

In order to investigate the difference between the bacterial survival rate on the BMS and flat substrate in more detail, we numerically evaluated the temperature distribution on the substrate at a laser power of 47 mW, which is the lowest power required to stably generate a real air bubble and assemble dispersoids on a flat substrate. Figure 4 shows the results of the simulation performed by the finite element method with the simulation model shown in Supplementary Fig. 9a. For the flat substrate, near the region where the bacteria were assembled experimentally, a huge increase in temperature to a value higher than 100 °C was determined theoretically. On the other hand, the temperature of the assembly site on the BMS was found to be lower than 50 °C, as in the case of laser irradiation at 47 mW laser power. These results correspond with the experimental results shown in Fig. 2e; the bacteria on the BMS showed a higher survival rate than those on the flat substrate. The difference is caused by the decrease in the heat transfer to the stagnant area. On the flat substrate, the heat source is near the stagnant area where dispersoids assemble, so the heat due to the photothermal effect was conducted to the stagnant area. In contrast, on the BMS, the stagnant area is far away from the heat source, because the platinum thin film on the imitation bubble was irradiated with a defocused laser, so less heat was conducted to the stagnant area. Thus, thermal damage to assembled dispersoids was limited.

In addition, we investigated the mechanism of assembly due to laser-induced convection. The distribution of the convective velocity at a laser power of 27 mW was numerically evaluated. Figure 4c depicts the distribution of the convection velocity near the imitation bubble and Supplementary Fig. 10 shows that of the whole region. The cause of convection and the assembly mechanism can be attributed to the following process: first, the platinum thin film on the imitation bubble is heated by laser irradiation based on the photothermal effect. Second, the convective flow transports the dispersoids toward the imitation bubble. The convection around the imitation bubble is slow because of the no-slip boundary condition of the solid–liquid interface. We consider that such a stagnant region works as an assembly site. Finally, the bacteria in the dispersion liquid were assembled at the stagnant area. On the other hand, the temperature gradient would provide a large influence for the transport of bacteria toward imitation bubble, and there is a possibility that the thermophoresis effect^41,42^ would contribute to assemble bacteria by BMS. In addition, considering an imaginary cylindrical area around the imitation bubble, as shown in Fig. 4e, f, from the numerical viewpoint, we estimated the number of bacteria transported close to the assembly site based on $N_{\mathrm{t}} = t_{{\mathrm{LI}}}{\int}_0^h {2\pi r_{{\mathrm{obs}}}\; j_{\mathit{r}}\left( z \right)dz}$, where *t*_LI_ is the laser irradiation time (=300 s), *r*_obs_ is the radius of the cylinder (=150 μm) in Fig. 4e, f corresponding to an observation area, *h* is the height of the cylinder, and $j_{\mathit{r}}\left( z \right) = - C_{{\mathrm{bac}}}u_{\mathit{r}}(z)$ is the flux of the bacteria flowing into the imaginary cylinder across its side (*C*_bac_ = 1.43 × 10^8^ cells/mL is the concentration of bacteria, $u_{\mathit{r}}\left( z \right)$ is the *r*-component convective velocity at *r*_obs_ shown in Fig. 4d). When $h = 100\,{\mathrm{\mu m}}$, the number of bacteria transported is *N*_*t*_ = 54,900, which is of the same order of magnitude as the experimentally determined number of assembled bacteria (40,000 cells) at a laser power of 27 mW. Thus, this assumption indicates that the bacteria dispersed over a distance ranging up to *z* = 100 µm were assembled.

**Fig. 5:**
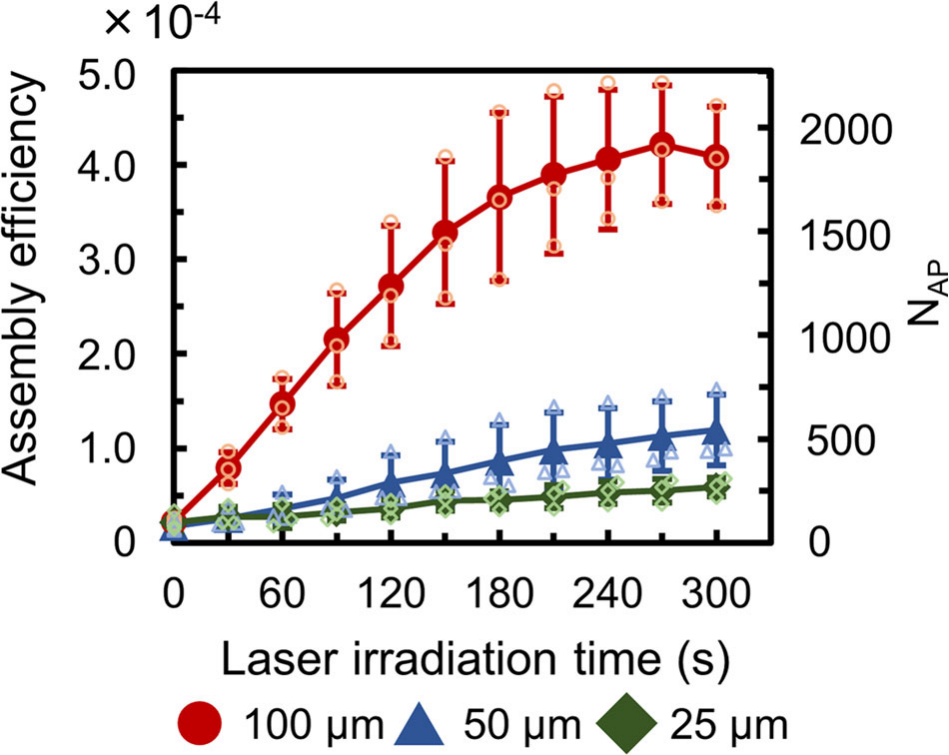
Time dependence of the assembly efficiency and the number of assembled particles (*N*_AP_) with each imitation bubble size. The dispersoids are polystyrene particles (diameter is 1 µm) and the concentration of particles is 4.55 × 10^7^ particles/mL (*n* = 3 independent experiments). The error bars represent the standard deviation. Individual values are shown as small plots.

Finally, in order to investigate the effect of imitation bubble size, experiments for light-induced assembly of polystyrene particles (diameter: 1 µm, concentration: 4.55 × 10^7^ particles/mL) with imitation bubble (diameter: 100, 50, and 25 µm) were performed and the time dependence of assembly efficiency and number of assembled particles (*N*_AP_) were plotted in Fig. 5. In each assembly experiment, assembly efficiency and number of assembled dispersoisds were calculated from each 30 s snapshot of fluorescent image video for 300 s and laser power was set to the same laser intensity (power per unit area) on the top of imitation bubble (laser power: 30 mW at 100 µm, 7.5 mW at 50 µm, and 1.9 mW at 25 µm). Particularly, Fig. 5 shows assembly efficiency and *N*_AP_ was higher for large imitation bubble size that can trap many dispersoids since stagnant area gets larger for the larger imitation bubble. Therefore, 100 µm imitation bubble was optimal size for light-induced assembly on BMS as far as we investigated. In addition, assembly efficiency of 100 µm imitation bubble was saturated after 270 s. This indicates that the stagnant area between imitation bubble and substrate was filled with assembled particles. Therefore, 300 s laser irradiation was long enough for assembly with 100 µm imitation bubble.

## Discussion

In this study, we succeeded in achieving large-scale and damage-free light-induced assembly of a vast number of microbial cells at the solid–liquid interface on a substrate without bubble generation under laser irradiation using a designed bubble-mimetic substrate consisting of a submillimeter polymer particle as an imitation bubble. As an example, 10^4^–10^5^ cells of *L. casei* were assembled on the BMS within a few minutes, the survival rate was found to be higher than 95% even at high laser powers and different bacterial concentrations. These results are attributed to the decrease in heat conduction to the stagnant area, which serves as the assembly area on this substrate.

This method can be extended to unconventional biological analysis for the evaluation of the functions of not only intestinal bacteria but also assembled beneficial bacteria (e.g., electricity-producing bacteria and ethanol-producing bacteria) or physicochemical interactions of dense bacterial colonies. In the future, the method based on the clarified mechanism described herein may be used for evaluating the symbiotic mechanism of various types of beneficial bacteria, pathogenic properties of harmful bacteria or viruses, and the effect of drugs on bacteria.

## Methods

### Sample preparation

A lactic acid bacterium, *L. casei* (average long axis: 1.22 µm, average short axis: 0.51 µm), *S. aureus* (average diameter: 0.91 µm), and polystyrene particles (diameter: 1 µm) with COOH modification (Fluoresbrite Carboxylate Microspheres (2.5% Solids-Latex), 1.0 μm-YG, Polysciences, USA) were used as the dispersoid. The size of *L. casei* was determined from the scanning electron microscopy image (JSM-IT100, JEOL Ltd., Japan) shown in Supplementary Fig. 1. Ultrapure water was used as the solvent. The concentration of *L. casei* and *S. aureus* were estimated by the cultivation method. The concentration of *L. casei* was determined thrice over 72 h with pour culture and that of *S. aureus* was determined thrice over 24 h with Petrifilm (Petrifilm; 3 M Health Care, USA), and the average value was used. Further, *L. casei* and *S. aureus* were stained with SYTO9 (a green fluorescent dye) and PI (a red fluorescent dye) (LIVE/DEAD® BacLightTM Bacterial Viability Kit for microscopy, Invitrogen, USA).

### Preparation of the bubble-mimetic substrate

First, ethanol (360678-5 G, Sigma Aldrich, USA) was dropped on a glass-bottom dish (3911-035, IWAKI, Japan) and the glass-bottom dish was cleaned ultrasonically (MCD-2, ASONE, Japan) for 10 min at 30 kHz and 40 kHz. Next, after mixing 10 µL of dispersion liquid of polystyrene particles (diameter: 100 µm, concentration: 4.64 × 10^4^ particles/mL) with COOH modification (MPT-01-02-105-10; Micromod Partikeltechnologie GmbH, Germany), 287 µL of ultrapure water, and 3 µL of 10 times diluted 3-(trimethoxysilyl)propylmethacrylate (440159-500 ML; Sigma Aldrich, USA) as a silane coupling agent, and the mixture was dropped on the glass-bottom dish and dried naturally in a draft chamber. Next, 100 µL of ultrapure water was dropped on the substrate, which was left undisturbed for 5 min; subsequently, water was removed using Prowipe (Elleair, Japan) and the substrate was dried naturally. Finally, a platinum thin film (thickness: 10 nm) or gold thin film (thickness: 10 nm) were formed on the substrate by ion sputtering (MC1000; Hitachi, Japan).

### Optical setup

The optical system used in this paper is shown in Supplementary Fig. 2a. An inverted optical microscope (Eclipse Ti-U; Nikon, Japan) was used for the light-induced assembly of bacteria by laser irradiation with a back port adapter (MMS-2L-800/1064; Sigma Koki, Japan). A 100 µL of the sample dispersion liquid was dropped on the substrate, and the substrate was placed on the stage of the microscope. Then, a non-ionic surfactant, polyoxyethylene sorbitan monolaurate (T20; Wako Pure Chemical Industries, Japan), was added to achieve a surfactant concentration of 9.04 × 10^−5^ M in the bacterial suspension. Thereafter, the near-infrared continuous wave laser of 1064 nm wavelength (FLS-1064-2000F, Sigma Koki, Japan) was focused using an objective (CFI S Plan Fluor ELWD 40XC, 0.6 NA) on the imitation bubble/glass substrate interface from the bottom. The laser spot diameter was 2.6 µm and the laser power was determined with a laser power meter (UP17O-H5 and TUNER; Gentec Electro-Optics, Canada). The halogen lamp to excite sample was turned off during laser irradiation in only *L. casei* assembly experiment. After 300 s of laser irradiation, fluorescent images were recorded. The fluorescent image movie was also recorded during laser irradiation to investigate time dependence of assembly efficiency and the number of assembled dispersoids. In order to observe the imitation bubble from side, another optical system shown in Supplementary Fig. 2b was used; it is a microscope based on OTKB/M (THORLABS, United States) and an objective lenses (CFI S Plan Fluor ELWD 20XC, 0.45 NA for Fig. 1b and OLYMPUS SLMPLN50x 0.35 NA for Fig. 1c).

### Estimation of the number of bacteria in the assembly

The number of assembled dispersoids was estimated from fluorescent images by using software (NIS-Elements Analysis; Nikon, Japan). The fluorescence intensity of the bacteria at the edge of the fluorescent image was determined to be a standard value; the fluorescent area over the standard value was measured in the whole observation area, and *N*_AB_ was estimated by dividing the total fluorescent area by the area of an individual dispersoid determined from the SEM image. Survival rate is the ratio of the number of the live bacteria to the total number of bacteria calculated by the previous method. In the case of the flat substrate, *N*_AB_ was estimated as reported in a previous study^31^ and the survival rate was calculated based on it.

### Finite element method simulation for light-induced assembly with BMS

Distributions of the convective velocity and temperature were calculated with 2D rotational symmetry condition based on finite element method using COMSOL Multiphysics (COMSOL AB, Sweden). Supplementary Fig. 9a shows the 2D simulation model of the bubble-mimetic substrate. The Navier–Stokes equation, mass continuity equation, and energy equation were used. The velocity was solved for the liquid region (water), whereas the temperature was solved for the entire region including the substrate (glass) and the imitation bubble (polystyrene) or the bubble (air). In the case of the bubble-mimetic substrate, we assumed that the laser focused on the glass substrate was defocused at 80 µm from the glass substrate. We assumed that the region indicated by red line (spherical segment on top of the imitation bubble) shown in Supplementary Fig. 9a generates heat according to, *Q*_a_ = *P·A*/*S*, where *P* is the laser power (=47 mW in Fig. 4b, 27 mW in Fig. 4c), *A* is the absorptance (=0.5; it was determined by electromagnetic simulation described below), and *S* is the surface area of the spherical segment (=6283.2 µm^2^). In the case of the flat substrate, a point heat source with *Q*_p_ = *P·A* was assumed, because the laser was focused on the substrate. We assumed a no-slip and slip boundary conditions for the solid/liquid and air/liquid interfaces. In addition, we assumed that the Marangoni effect at the air/liquid interface depends on the experimental concentration of the surfactant^31^. The mesh size was nonuniform; the maximum size was 296 μm and the minimum size was 1 μm for the center region.

### Finite difference time domain simulation for calculation of the absorptance

The absorptance was calculated based on finite difference time domain simulations (FDTD) using Lumerical FDTD Solutions (the simulation model is shown in Supplementary Fig. 9b). We assumed that the flat platinum thin film (thickness: 10 nm) on the polystyrene substrate was irradiated by a plane wave. The boundary condition was perfectly matched layer (PML) for the wall perpendicular to light-propagation direction, and a periodic boundary condition for the other walls. As a result, the absorptance was determined to be 0.5 (see the absorption spectrum in Supplementary Fig. 9c). The mesh size was 1 nm.

### Statics and reproducibility

The error bars in each plots show standard deviation and each plot show average to *n* = 3 samples. The experiments were performed three times by using the different bubble-mimetic substrate.

### Reporting summary

Further information on research design is available in the Nature Research Reporting Summary linked to this article.

## Supplementary information

Supplementary Information

Description of Additional Supplementary Files

Supplementary Movie 1

Supplementary Data 1

Reporting Summary

## Data Availability

All data needed to evaluate the conclusions in the paper are present in the paper and/or the Supplementary Materials. Additional data related to this paper may be available from the corresponding authors on reasonable request.
