# Supplementary material for: Damage-free light-induced assembly of intestinal bacteria with a bubble-mimetic substrate
Source: Commun Biol. 2021 Mar 22;4:385. doi: 10.1038/s42003-021-01807-w (PMC7985151; doi:10.1038/s42003-021-01807-w)
Supplement: Supplementary file 1 — Supplementary Information [file 42003_2021_1807_MOESM1_ESM.pdf]

SUPPLEMENTARY INFORMATION

Damage-free Light-induced Assembly of Intestinal Bacteria with a Bubble-mimetic Substrate

Kota Hayashi[1,2,3], Yasuyuki Yamamoto[1,2,3], Mamoru Tamura[1,2], Shiho Tokonami[2,3,*,#], and Takuya Iida[1,2,*,#]

[1]Department of Physical Science, Graduate School of Science, Osaka Prefecture University, 1-2, Gakuen-cho, Naka-ku, Sakai, Osaka 599-8570, Japan

[2]Research Institute for Light-induced Acceleration System (RILACS), Osaka Prefecture University, 1-2, Gakuen-cho, Naka-ku, Sakai, Osaka 599-8570, Japan.

[3]Department of Applied Chemistry, Graduate School of Engineering, Osaka Prefecture University, 1-2, Gakuen-cho, Naka-ku, Sakai, Osaka 599-8570, Japan

*Correspondence to: t-iida@p.s.osakafu-u.ac.jp (T.I.); tokonami@chem.osakafu-u.ac.jp (S.T.)

[#]These authors contributed equally to this work.

Supplementary Figures:

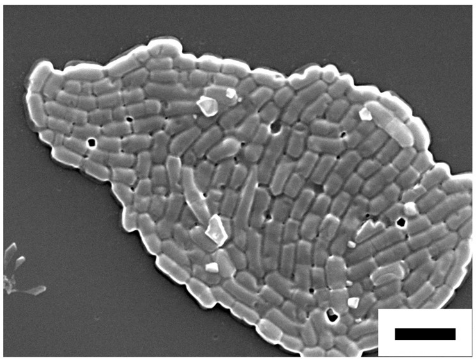

Supplementary Figure 1. Scanning electron microscopy image of *Lactobacillus casei* (scale bar: 2 μm).

.

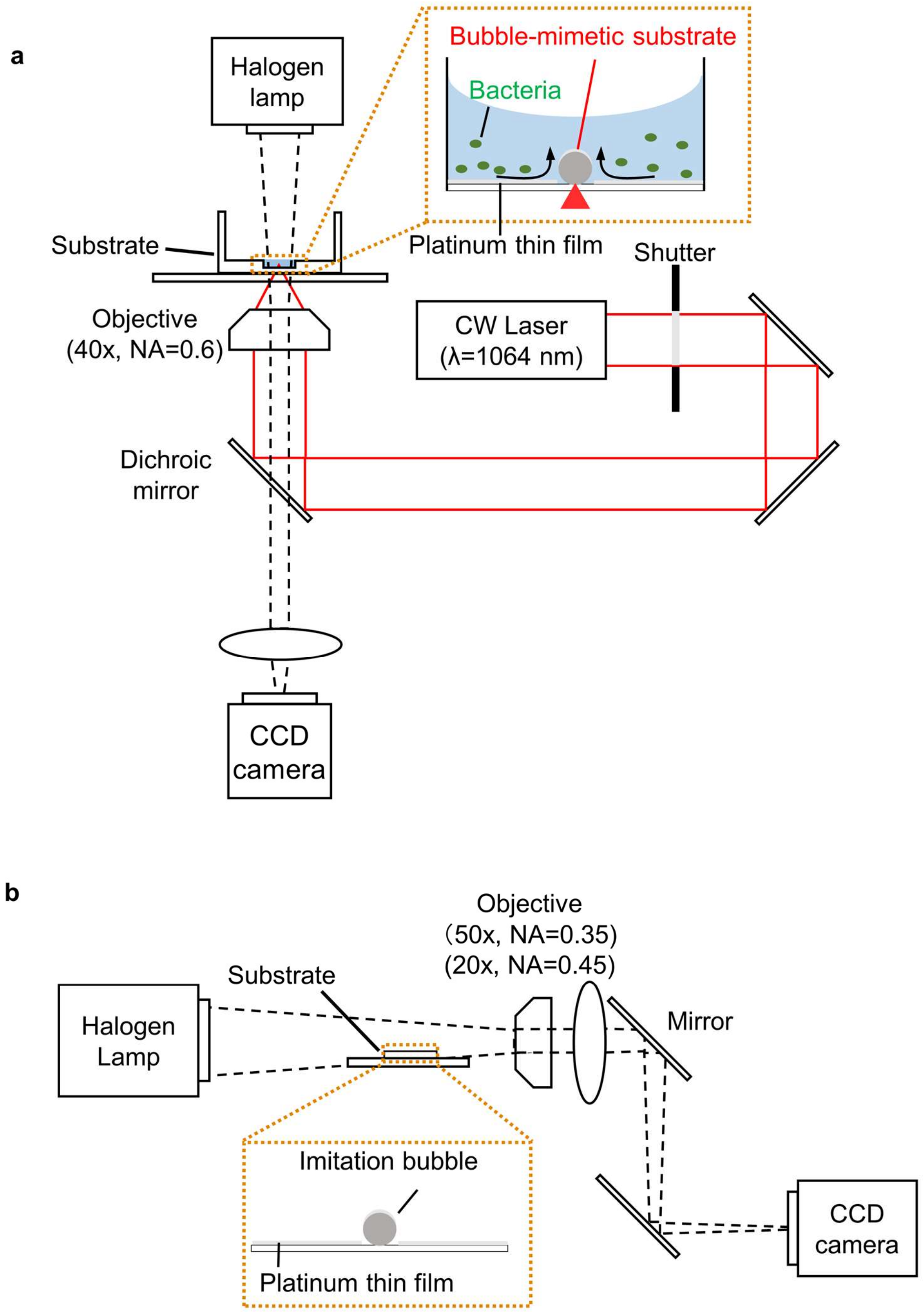

Supplementary Figure 2. A schematic diagram of the optical system for experiment. a, The optical system for damage-free light-induced assembly. **b**, The optical system for observation of substrate from side.

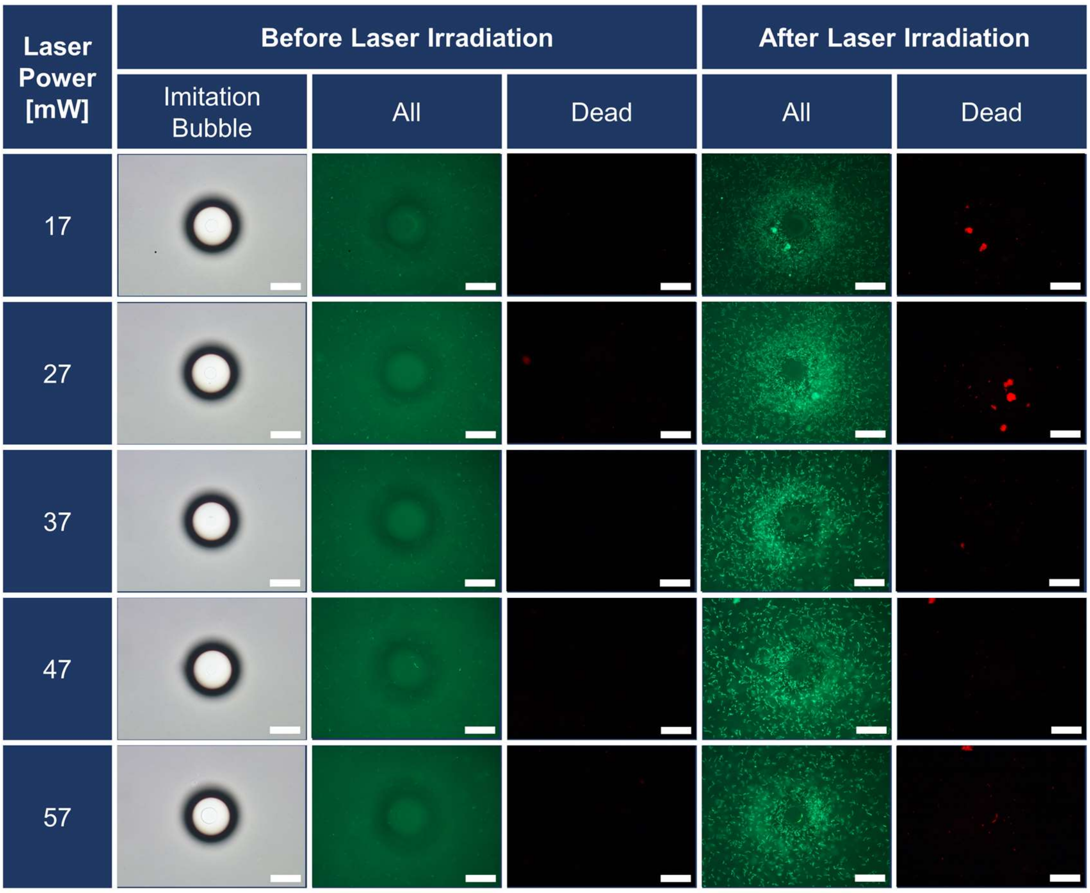

Supplementary Figure 3. Optical transmission images and florescent images before and after light-induced assembly (LIA) of *L. casei* with BMS. *L. casei* concentration is 1.43×10^{8} cells/mL (all the scale bars: 50 μm). Laser irradiation time was 300 s for LIA.

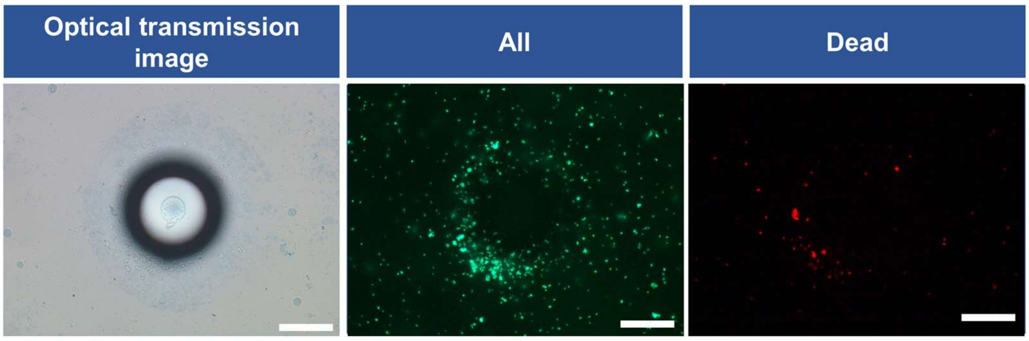

Supplementary Figure 4. Optical transmission images and florescent images after LIA of *Staphylococcus aureus* with BMS formed gold thin film. *Staphylococcus aureus* concentration is 2.67×10^{7} cells/mL (the length of scale bar: 50 μm). Here, laser irradiation time is 300 s and laser power is 27 mW.

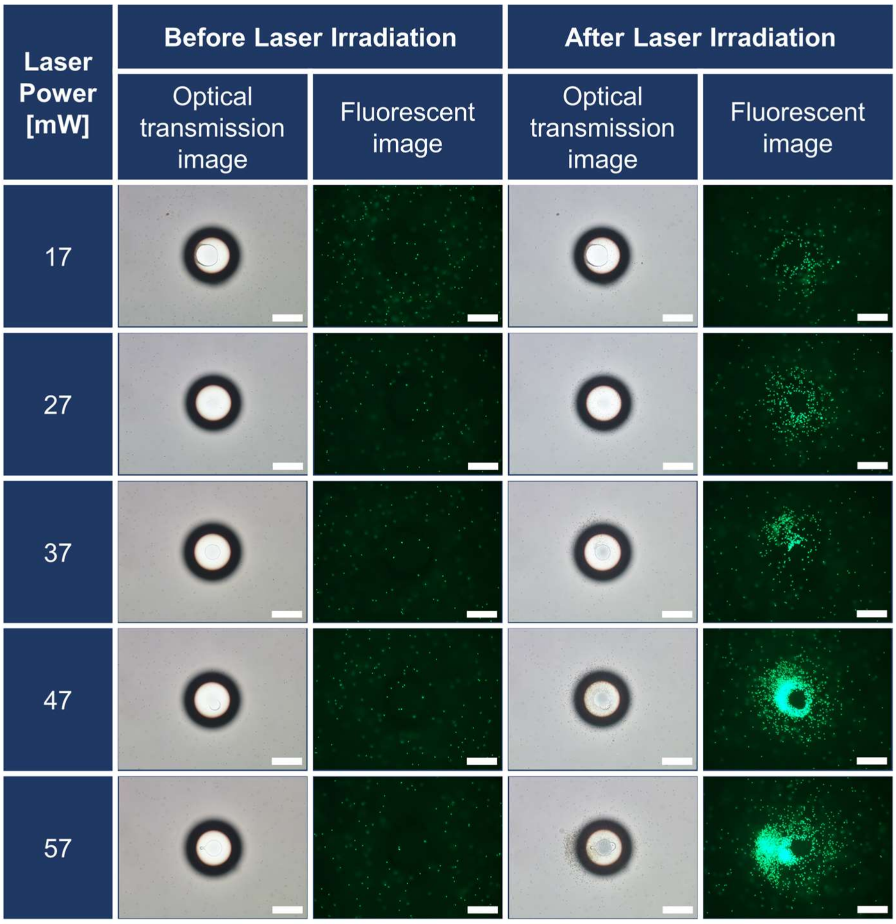

Supplementary Figure 5. Optical transmission images and florescent images before and after LIA of polystyrene particles. Diameter of each polystyrene particle is 1 μm and concentration in dispersion liquid of polystyrene particles is 4.55×10^7 particles/mL (the length of scale bar: 50 μm). Here, laser irradiation time is 300 s.

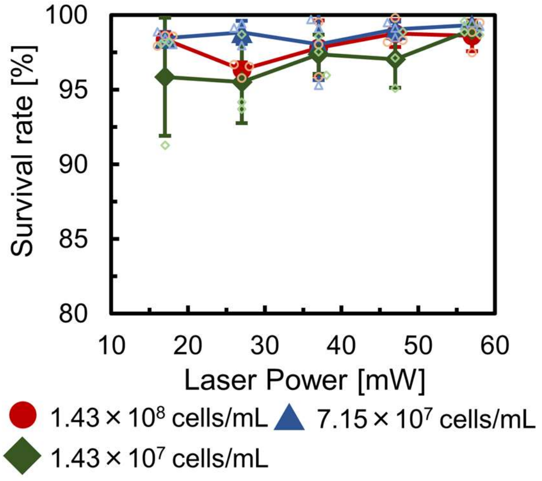

Supplementary Figure 6. Survival rate dependence on laser power with each concentration on bubble-mimetic substrate. The error bars represent the standard deviation (n = 3 independent experiments). Individual values are shown as small plots.

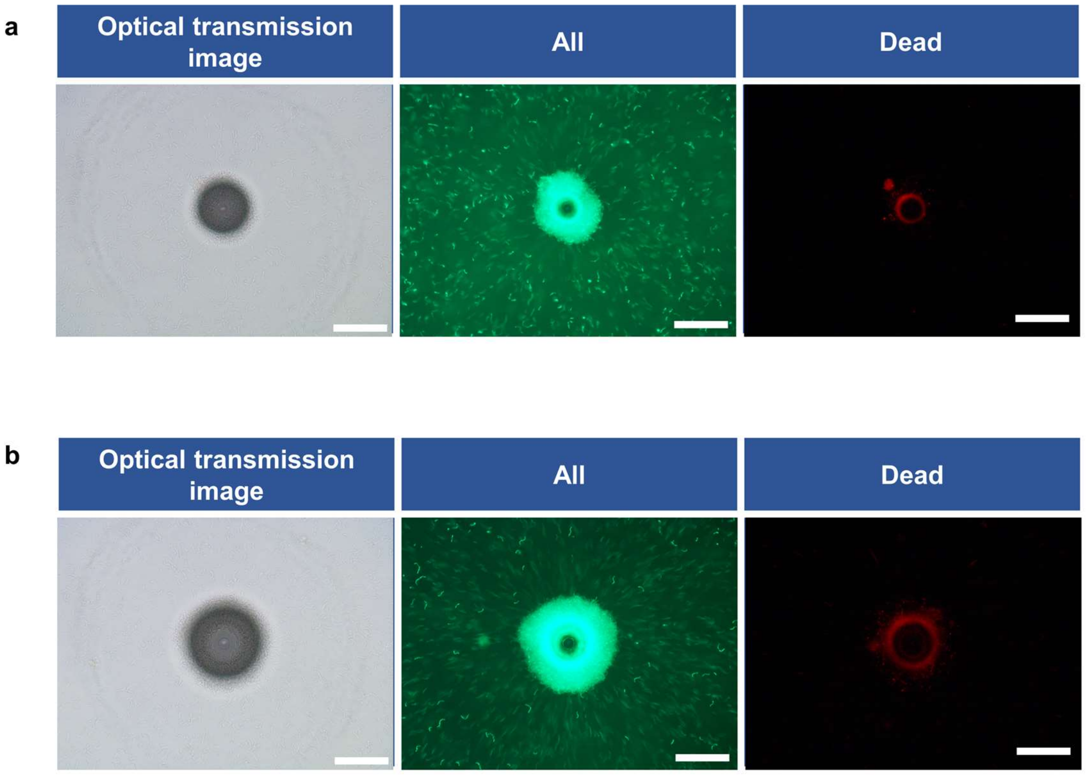

Supplementary Figure 7. Optical transmission images and florescent images after LIA of *L. casei* with flat substrate. Here, laser irradiation time is 300 s and concentration of *L.casei* is 2.38×10^{8} cells/mL. **a,** Laser power is 47 mW, **b,** Laser power is 97mW (scale bar :50 µm).

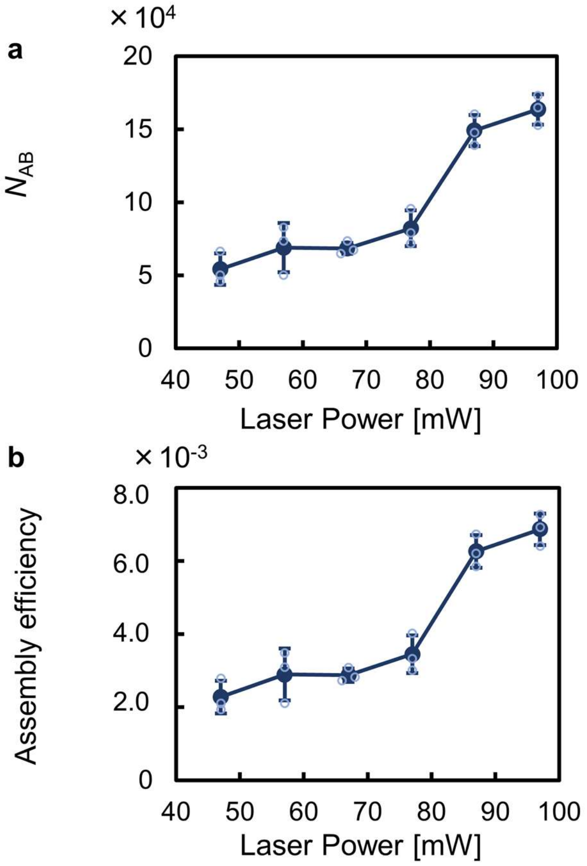

Supplementary Figure 8. Relationship between the laser power and number of assembled bacteria (N_{AB}) or assembly efficiency at each bacterial concentration studied on flat substrate. a, N_{AB} dependence on laser power (n = 3 independent experiments), **b**, assembly efficiency of bacteria dependence on laser power (n = 3 independent experiments). The error bars represent the standard deviation. Individual values are shown as small plots.

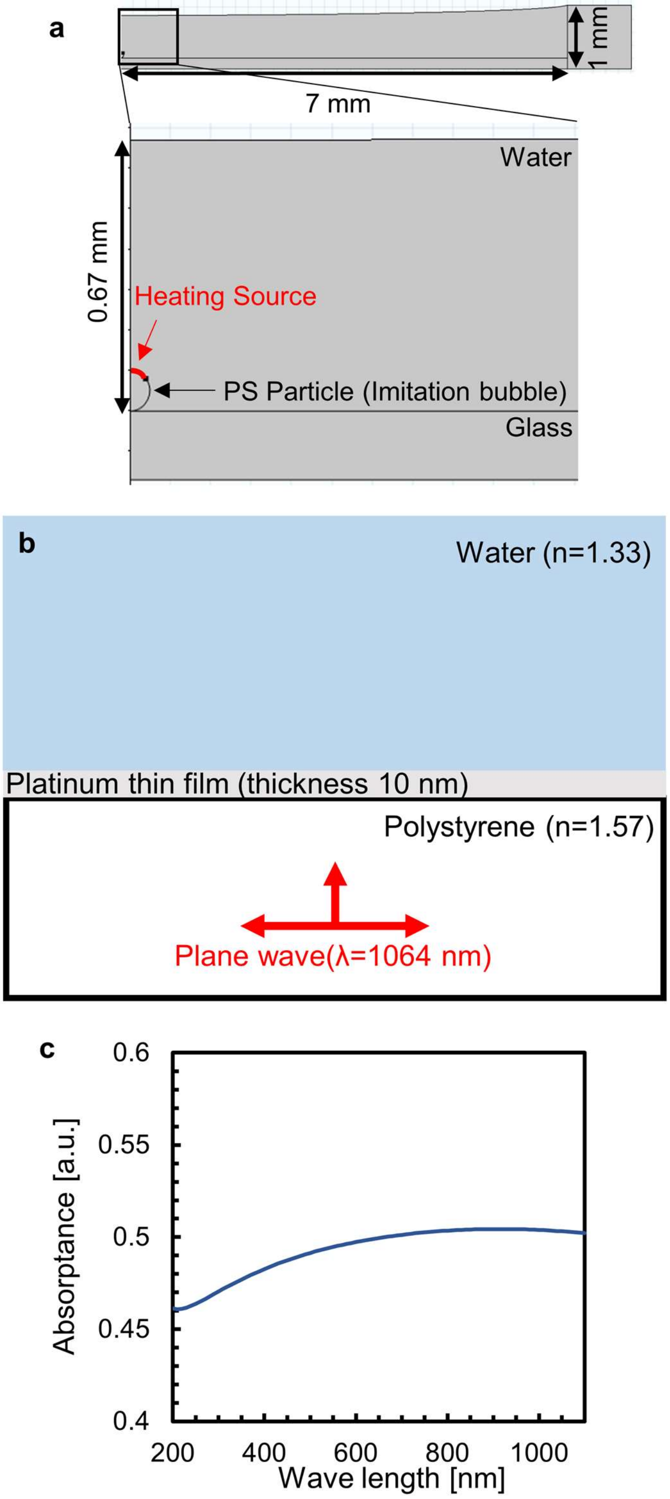

Supplementary Figure 9. Calculation model for LIA with BMS. a, Finite element method and **b** finite difference time domain simulation. **c**, Absorptance spectrum based on the finite difference time domain simulation.

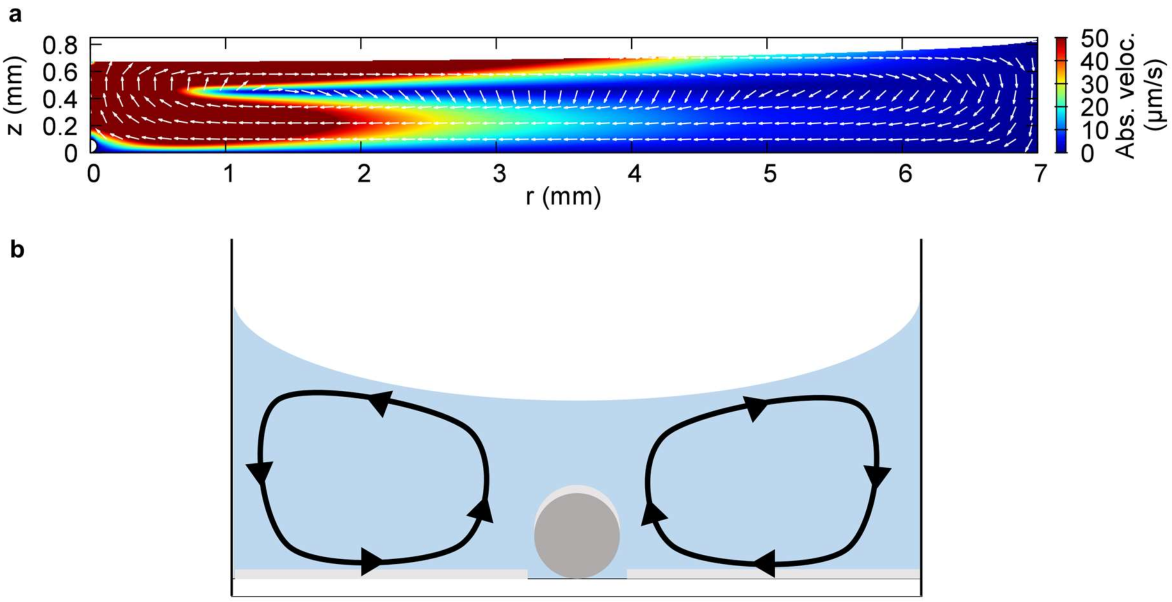

Supplementary Figure 10. The simulated result of all area on substrate. a, The distribution of absolute convective velocity and **b,** schematic diagram of convection.
