# Supplementary material for: Damage-free light-induced assembly of intestinal bacteria with a bubble-mimetic substrate
Source: Commun Biol. 2021 Mar 22;4:385. doi: 10.1038/s42003-021-01807-w (PMC7985151; doi:10.1038/s42003-021-01807-w)
Supplement: Supplementary file 2 — Description of Additional Supplementary Files [file 42003_2021_1807_MOESM2_ESM.pdf]

**Description of Additional Supplementary File**

**File Name:** Supplementary Movie 1

**Description:** Video (20x) of light-induced assembly during 300 s on bubble-mimetic substrate irradiated by 27mW of laser power.

**File name:** Supplementary Data 1

**Description:** Source data for the charts and graphs in the main and supplementary figures.
